# Targeting cyclin D1 as a therapeutic approach for papillary thyroid carcinoma

**DOI:** 10.3389/fonc.2023.1145082

**Published:** 2023-06-22

**Authors:** Wei Cai, Lin-Zhen Shu, Ding-Jie Liu, Lv Zhou, Meng-Meng Wang, Huan Deng

**Affiliations:** ^1^ Department of Pathology, The Fourth Affiliated Hospital of Nanchang University, Nanchang, China; ^2^ Medical College, Nanchang University, Nanchang, China; ^3^ Zhuhai Interventional Medical Center, Zhuhai Precision Medical Center, Zhuhai People’s Hospital, Zhuhai Hospital Affiliated with Jinan University, Zhuhai, China

**Keywords:** cell cycle, cyclin D1, CCND1, papillary thyroid carcinoma, pathogenesis

## Abstract

Cyclin D1 functions as a mitogenic sensor that specifically binds to CDK4/6, thereby integrating external mitogenic inputs and cell cycle progression. Cyclin D1 interacts with transcription factors and regulates various important cellular processes, including differentiation, proliferation, apoptosis, and DNA repair. Therefore, its dysregulation contributes to carcinogenesis. Cyclin D1 is highly expressed in papillary thyroid carcinoma (PTC). However, the particular cellular mechanisms through which abnormal cyclin D1 expression causes PTC are poorly understood. Unveiling the regulatory mechanisms of cyclin D1 and its function in PTC may help determine clinically effective strategies, and open up better opportunities for further research, leading to the development of novel PTC regimens that are clinically effective. This review explores the mechanisms underlying cyclin D1 overexpression in PTC. Furthermore, we discuss the role of cyclin D1 in PTC tumorigenesis *via* its interactions with other regulatory elements. Finally, recent progress in the development of therapeutic options targeting cyclin D1 in PTC is examined and summarized.

## Introduction

The global incidence of thyroid cancer has increased considerably over the past few decades (1). Papillary thyroid carcinoma (PTC), derived from thyroid follicular cells, is the most common endocrine malignancy, accounting for up to 80% of all thyroid carcinomas (2, 3). Early-stage of PTC can be detected using ultrasonography and fine-needle biopsy. However, the typically indolent nature of PTC often leads to a delay in diagnosis that may substantially worsen the course of the disease (4). Consequently, the incidence of advanced large-sized PTCs has increased (2).

Despite the overall survival of patients with PTC being high, lymph node metastasis is observed during diagnosis in almost 36% of cases (5, 6). The biology of PTC is extremely diverse, ranging from non-progressive lesions to aggressive metastatic carcinomas. Although traditional thyroidectomy combined with radioactive iodine therapy is recommended as the first-line treatment for PTC, it is often incurable, leading to a high recurrence rate (4). Recurrence in the neck is a serious complication that may be considered as a sign indicating a potentially lethal outcome (7, 8). Patients at low risk for PTC death may still experience significant or even disastrous morbidities, including invasion of the paratracheal regions, large cervical vessels, and recurrent laryngeal nerves (3, 9). Thus, a deeper understanding of the various signaling pathways involved in PTC progression may help facilitate the development of effective molecular drugs.

Cyclins and their catalytic partners, cyclin-dependent kinases (CDKs), which regulate the eukaryotic cell cycle, are considered as promising candidates for primary involvement in oncogenesis (10). The cyclin D1 gene (*CCND1*), located on human chromosome 11q13, is an established oncogene (11). The dysregulation of cyclin D1 expression or CDK4/6 activation can directly lead to some of the hallmarks of cancer upregulation *via* causing proliferation or overriding of checkpoints (12, 13). In human cancer cell lines, cyclin D1 dysregulation contributes to cancer development *via* its interactions with more than 100 proteins (14). Cyclin D1 overexpression leads to dysregulated cell proliferation, as well as malignant tumor transformation and development, including PTC (15–17). In this review, we address the role of cyclin D1 in PTC and discuss the potential therapeutics of cyclin D1-based treatments.

## Cyclin D1 in normal cells

Unique cyclins accumulate at various stages of the cell cycle, contributing to transcription and protein degradation inhibition (18). The synthesis of a single cyclin and the subsequent activation of CDK form active heterodimeric complexes at specific cell cycle phases (13, 19) that coordinate DNA replication and cell division (20). In normal cells, the processes of expression, activation, distribution, stabilization, and degradation of cyclin D1 are strictly regulated by the on/off signal response of mitosis (21, 22). In contrast to cancer cells, normal cells require extracellular signaling to proliferate *via* the binding of extracellular matrix components to adhesion receptors (integrins) and growth factor receptors (receptor tyrosine kinases). Thus, integrin and growth factor signaling pathways ensure that cell proliferation is restricted to cells exposed to appropriate chemical and physical cues (19, 23, 24).

**Figure 1:**
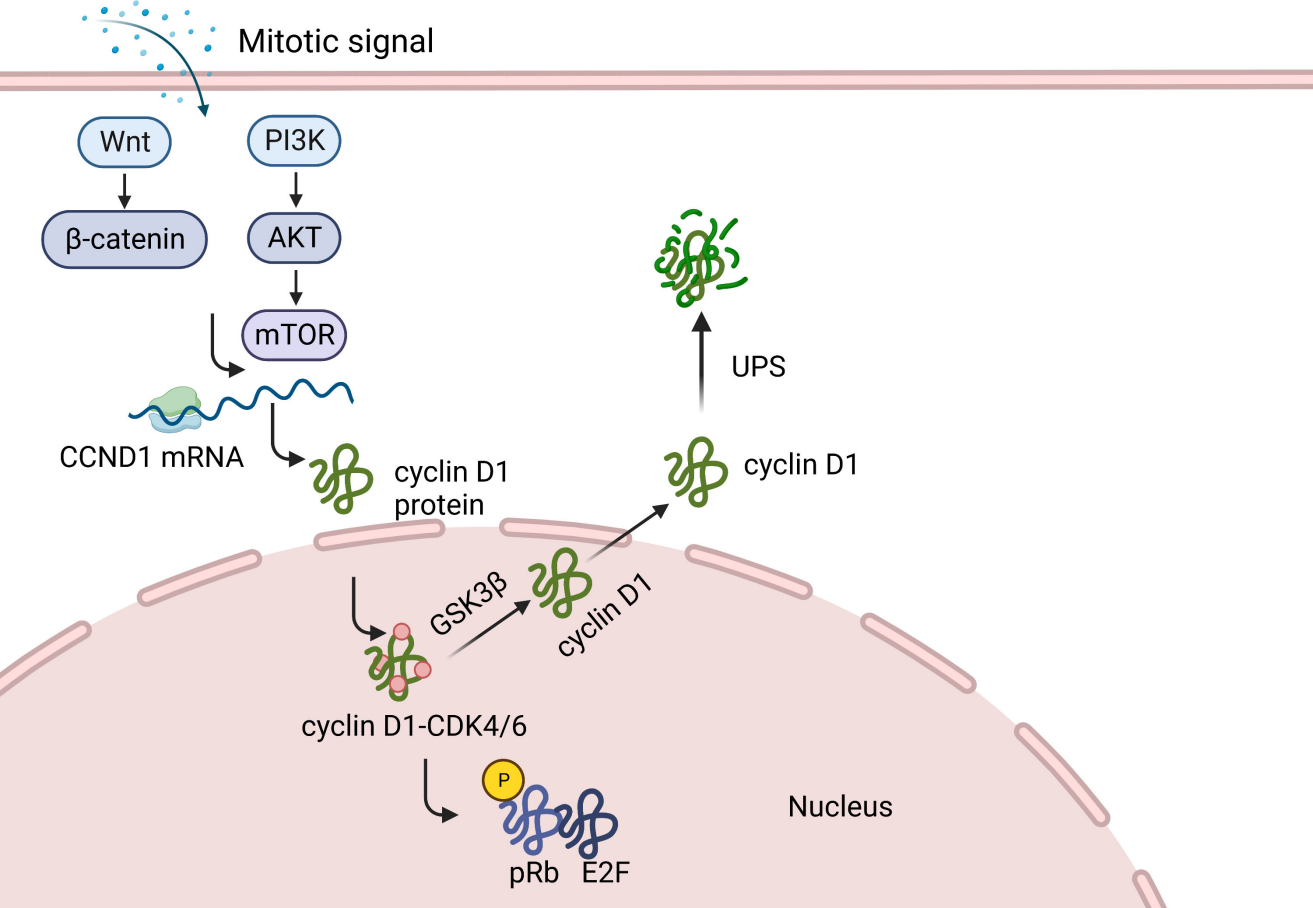
Function of cyclin D1 in normal cells. When mitogenic stimulation induces quiescent cells to enter G1, the synthesis of CCND1 mRNA and cyclin D1 protein begins. Cyclin D1, by forming different heterodimeric complexes with CDK4/6, phosphorylates and inactivates retinoblastoma protein (pRB), causing the expression of a subset of proliferation associated E2F target genes. This, in turn activate some genes in response to the G1 phase progression, thereby initiating DNA replication and regulating the transcription of specific cell proliferation genes. Cyclin D1 is then transported to the cytoplasm and degraded by UPS.

The G1 phase represents the stage at which cells respond to extracellular signals (25). Cell cycle regulation requires sustained activation of signaling pathways, such as the ERK pathway. Continuous stimulation of mitogen-activated protein kinase (MAPK) is a common requirement for cyclin D1 expression in the G1 phase and cell cycle re-entry (**Figure 1**) (22, 26, 27). Syntheses of CCND1 mRNA and cyclin D1 protein begins when mitogenic stimulation induces quiescent cells to enter the G1 phase (28). Activation of the RAS-mediated signaling cascade and phosphoinositide-3-kinase (PI3K)/AKT induces CCND1 translation and reduces cyclin D1 degradation (23, 28–30). Growth factor signaling accelerates the formation of cyclin D1-CDK4/6 dipolymer *via* a Ras-dependent pathway (30). Cyclin D1-CDK4/6 phosphorylates and inactivates retinoblastoma proteins (pRB), resulting in the expression of a subset of proliferation-associated E2F target genes in response to G1 progression (13, 31). This process initiates DNA replication and regulates the transcription of specific cell proliferation genes (13). Cyclin D1 levels increase from early to late G1 phase and then decrease during the S phase (32–34). From G1 to S phase, cyclin D1 is exported to the cytoplasm and degraded by the ubiquitin-proteasome system (UPS) *via* the phosphorylation of a threonine residue (Thr286) at the carboxyl terminus (28, 35, 36). Studies have shown that Thr286 is mutated in a variety of cancers and that, in animal models, the production of mutant alleles generates spontaneous tumors, demonstrating the tumorigenic potential of cyclin D1 (35, 37, 38). The SCF complex, consisting of an S-phase kinase-associated protein (SKP1) adaptor, CUL1 scaffold and F-box proteins, facilitates the ubiquitination of phosphorylated cyclin D1 in the cytoplasm (33, 39–41). There are at least four E3 ubiquitin ligases in the F-box family, three of which (SKP2, FBXW8, and FBXO4) participate in the normal cell cycle, whereas the fourth (FBXO31) is involved in genotoxic stress (33, 39–41). Cyclin D1 degradation occurs *via* its direct interaction with the FBXO31 F-box motif and its phosphorylation at Thr286 (40). Cyclin D1-specific E3 ubiquitin ligase mutations result in the accumulation of cyclin D1 in cancer cells (41, 42).

## Cyclin D1 as a human oncogene

In line with high *CCND1* expression in solid cancers, cyclin D1 is more frequently dysregulated than cyclin D2 or D3 (43–46). There are several explanations for upregulation of cyclin D1, including gene amplification, chromosomal rearrangement, increased gene transcription and protein translation, decreased miRNA expression, and ubiquitination-mediated protein degradation inefficiency or loss (11, 32). Chromosomal translocation in the mantle cell lymphoma (MCL) places the *CCND1* under the control of the immunoglobulin heavy chain enhancer, resulting in the abnormal accumulation of cyclin D1 in tumor cells (46–48). In breast cancer, the cyclin D1 overexpression may be attributed to an increase in *CCND1* copy numbers (49, 50). In addition, the positive association between cyclin D1 expression and tumor progression has been validated in different cancers, including lung adenocarcinoma, head and neck squamous cell carcinoma, and esophageal squamous cell carcinoma (ESCC) (51–53).

## Cyclin D1 and PTC

### Dysregulation of cyclin D1 in PTC

Tumor markers constitute the principle of rationalizing the complexity of occurrence and development of tumor diseases that includes, maintaining proliferation signals, evading growth inhibitors, resisting cell death, achieving replication immortality, inducing angiogenesis, and activating invasion and metastasis (12).

A high prevalence of cyclin D1 overexpression in PTC has been demonstrated by several studies (54–60). It is possible that various forms of primary clonal damage may secondarily lead to the dysregulation of cyclin D1, thus providing alternative pathways for cells to develop similar tumor characteristics. Although cyclin D1 is aberrantly overexpressed in PTC, neither translocation nor gene amplification has been reported (61, 62), indicating that pathogenic activation of cyclin D1 may occur *via* additional mechanisms, including transcriptional and post-transcriptional dysregulation (63, 64). Jeon S et al., showed that CCND1 mRNA levels in PTC are higher than those in benign diseases (65). It is hypothesized that overexpression of cyclin D1 in PTC with high cyclin D1 levels, may not be due to *CCND1* amplification, but rather to deregulation of a trans-acting inducer of expression or mRNA degradation machinery.

The expression of cyclin D1 can be regulated by miRNAs (57, 66, 67) that negatively modulate gene expression by binding to the 3′-UTRs of targeted mRNA (68). MiR-211 has been found to bind directly to cyclin D1 mRNA and inhibit its expression in cancers (69, 70). Molecular evidence obtained from clinical PTC samples has revealed a significant inverse correlation between MiR-195 and *CCND1 (* 71). A dual-luciferase reporter assay revealed that co-transfection of MiR-195 inhibits the activity of the luciferase reporter with wild-type 3′-UTR of CCND1, resulting in a consistent negative correlation with the above clinical samples (71). In addition, MiR-195 suppresses the Wnt/β-catenin pathway in PTC, thus significantly reducing the protein levels (cyclin D1) involved in this pathway (71). Targeting MiR-195 may reverse cyclin D1-mediated cellular effects, including cell proliferation, apoptosis, migration, and invasion in PTC (71). Paired box gene 8 (*PAX8*) plays a critical role in thyroid development (72). MiR-144-3p binds to PAX8, that indirectly regulates the expression of cyclin D1 in PTC, thereby promoting cell cycle progression (72). MiR-1256 inhibits PTC cell growth and induces G0/G1 phase arrest. MiR-1256 is downregulated in PTC and its inhibitory effect on 5-hydroxy tryptamine receptor 3A (HTR3A) is dysregulated. High levels of HTR3A in PTC cells can partially eliminate the inhibitory effect of MiR-1256 on cyclin D1 expression (73). HTR3A knockdown significantly induces cell cycle arrest at the G0/G1 phase, hampering PTC cell proliferation (73). Lou et al., established the hsa_circ_0088494-miR-876-3p-CTNNB1/CCND1 axis using database analysis and found that it was associated with PTC carcinogenesis and progression (67).

Cyclin D1 is a target molecule in the aberrantly activated Wnt/β-catenin signaling pathway (74, 75). Peptidyl-prolyl cis-trans isomerase NIMA-interacting 1 (PIN1) contributes to the upregulation of cyclin D1, either directly or *via* the abnormal accumulation of β-catenin in tumor cells (76). PIN1 increases cyclin D1 expression, both transcriptionally and post-transcriptionally (77, 78). PIN1 interacts with c-Jun that is phosphorylated on Ser63/67-Pro motifs by activated oncogenic Ras/JNK, and subsequently boosts transcriptional activity of c-Jun to the cyclin D1 promoter (77). Moreover, PIN1 improves the protein stability and increases nuclear accumulation of cyclin D1 *via* heterodimerization (78). PIN1 inhibits nuclear export and protein degradation of β-catenin (79). Increased nuclear accumulation of β-catenin during Wnt/β-catenin cell signaling leads to enhanced transcriptional activity of β-catenin on downstream target genes, including *CCND1 (* 74). APC gene mutation is a common event in familial adenomatous polyposis coil (FAP)-related PTC, accompanied by nuclear β-catenin translocation (76, 80). In PTC cell lines, the formation of a destruction complex with APC is inhibited by PIN1 (76). Therefore, overexpression of PIN1 may be a factor of cyclin D1 upregulation and abnormal β-catenin expression during malignant thyroid gland transformation.

The constitutive activation of the MAPK pathway plays an important role in PTC development (81, 82). MAPK induces several mitotic and survival processes, including proliferation and protection from apoptosis *via* cell membrane substrate interactions and subsequent phosphorylation of transcription factors (83). The mutant genes involved encode the cell membrane-receptor tyrosine kinases, RET and NTRK1, as well as the intracellular signal transduction genes, BRAF and RAS (82). These mutations, which occur in approximately 70% of patients with PTC, are associated with specific clinical and biological tumor characteristics (16, 84–86). Important oncogenic mechanisms of the MAPK pathway in PTC include modulation of type 3 deiodinase (DIO3) to increase cyclin D1 expression (81). Nelfinavir (NFV), a MAPK/ERK pathway inhibitor, accelerates PTC cell arrest at the G0/G1 phase and downregulates cyclin D1 and CDK4 (87).

Cyclin D1 expression is also transcriptionally regulated by signal transducers and activators of transcription 3 (STAT3), known to be important for angiogenesis, tumorigenesis, and metastasis (88–92). STAT3 regulates the expression of cyclin D1 by binding to the *CCND1* promoter (93). Cyclin D1 mRNA levels are increased in tumor cells that express the STAT3 oncogenic variant (STAT3-C) or vSrc that constitutively phosphorylate STAT3 (93). Moreover, it has been proved that cyclin D1 is necessary for STAT3-C and vSrc-mediated anchorage-independent growth (93). In addition, both STAT3 and cyclin D1 protein levels in human PTC tissues are higher than those in adjacent non-tumorous thyroid tissues (88, 94).

The association between cyclin D1 and clinical parameters of PTC have been studied. In PTC, high cyclin D1 levels are associated with larger tumor size, intraglandular metastasis, extrathyroidal extension, lymph node metastasis, and aggressive behavior (17, 95–98). High cyclin D1 levels are linked to poor prognoses and high recurrence rates (56). These observations indicate that deregulated cyclin D1 expression may play an important role in determining the clinical course of PTC.

### Oncogenic consequences of cyclin D1 dysregulation in PTC

An essential characteristic of tumor cells is their ability to progress through proliferation limits set by tumor suppressors under conditions of cellular stress (18). In PTC, the dysregulation of cyclin D1 represents the initiation of sustained proliferative signaling acquisition and the malignant transformation of thyroid follicular cells. Activation of cyclin D1-CDK4/6 phosphorylates various substrates that regulate PTC cell proliferation, growth, migration, DNA repair, and centrosome duplication (11, 87). The cyclin D1-CDK4/6-Rb-E2F pathway regulates the G1 to S phase transition. During the S phase, cyclin D1 is exported to the cytoplasm and degraded (99). Dysregulation of these processes leads to the abnormal accumulation of cyclin D1 (100). Cell proliferation becomes independent of extracellular signals and bypasses cell cycle checkpoints responsible for ensuring genomic integrity (101, 102). Increased cyclin D1-CDK4/6 induces E2F-dependent transcription and promotes continuous proliferation of PTC cells by preventing cell cycle exit (18, 102–105).

Transcriptional regulation is one of the major non-catalytic functions of cyclin D1. Cyclin D1 affects the promoters of many genes by interacting with various transcription factors (106). Thrombospondin 1 (TSP-1), a multifunctional matricellular ECM and secreted protein (107), effectively inhibits tumorigenesis, cellular metastasis, and *in vivo* neovascularization (107, 108). Its promoter activity is repressed by cyclin D1 in a dose-dependent manner (108). Cyclin D1 inhibits TSP-1 transcription and mRNA expression and promotes cell migration (108, 109). Compared with those in normal thyroid tissues, TSP-1 mRNA levels are significantly lower in PTC tissues (110). The extrathyroidal infiltration of PTC is inversely correlated with TSP-1 expression (111). It has been shown that TSP-1 suppresses angiogenesis in PTC (17). These results further confirm that cyclin D1 promotes PTC carcinogenesis by downregulating TSP-1 expression.

Many studies have suggested the presence of a tight link between estrogen receptor-α (Erα) and cyclin D1 in PTC (112, 113). Cyclin D1 interacts with several members of the steroid hormone receptor superfamily and their co-regulators (63, 114). Cyclin D1 enhances the activity of Erα by interacting with its co-regulators, SRC1 (NCOA1) and AIB1 (NCOA3), in tumor cells (11). It also interacts with SRC1 and SRC3 to recruit additional transcriptional cofactors that increase the transcriptional activity of ER (115). Consequently, abnormally upregulated ER signaling leads to increased proliferation of PTC cell lines (112). These findings suggest that cyclin D1 overexpression promotes PTC development *via* the enhancement of Erα function.

## Cyclin D1-targeted treatments

Oncoproteins are appealing therapeutic targets due to their involvement in malignant cell behavior (116). Given its importance in PTC, cyclin D1 has come to be considered as an attractive drug target for clinical treatments. Fibroblasts, epithelial cells, and macrophages exhibit increased cellular adhesion and decreased motility in the absence of cyclin D1 (108, 117, 118). Previous studies have shown that suppression of cyclin D1 in PTC cells reduces cell proliferation, migration, and invasion (108, 119, 120).

**Figure 2:**
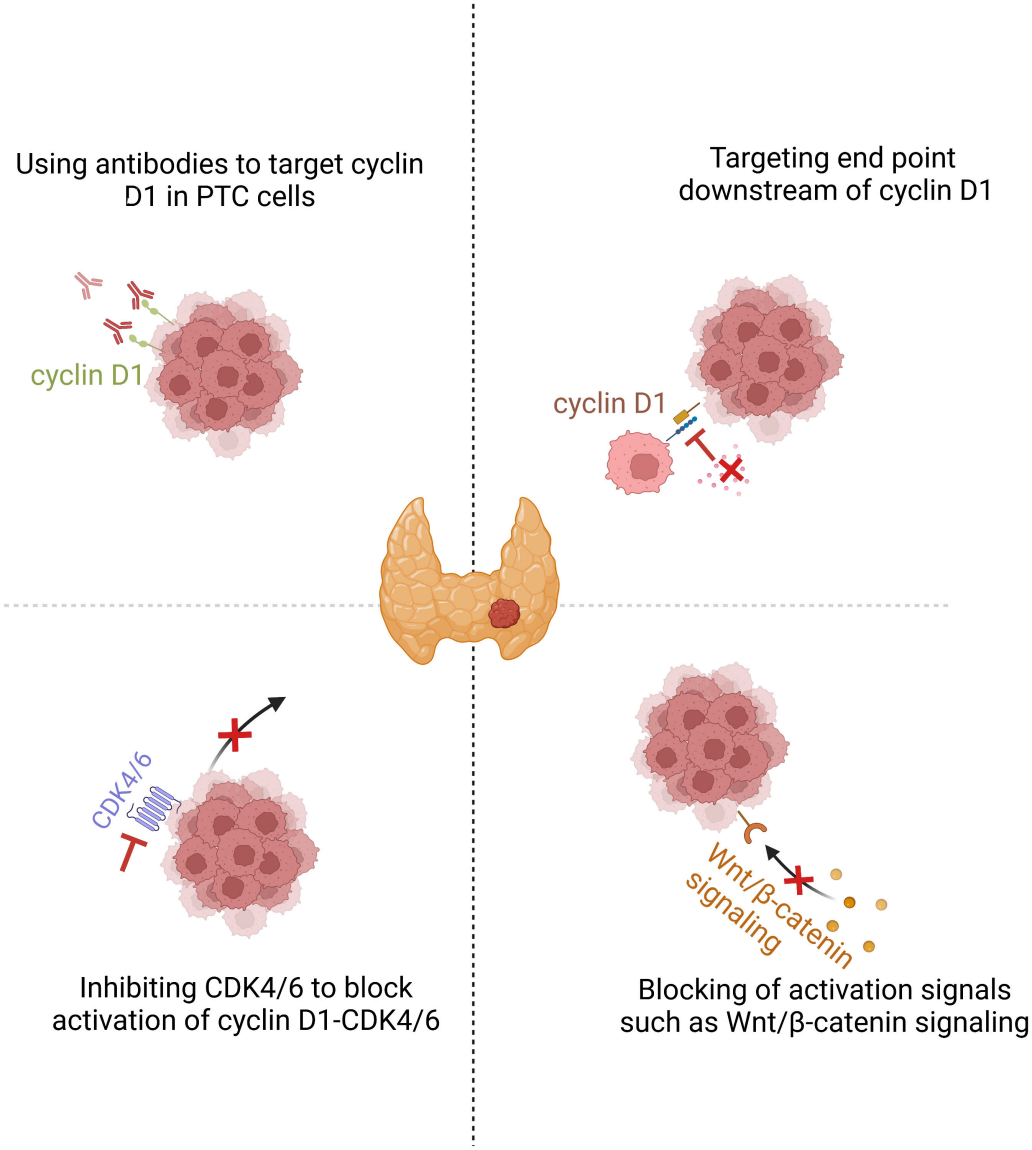
Cyclin D1 is a promising target in PTC. Downregulating cyclin D1 or inhibiting the end points of cyclin D1 action are two possible therapeutic approaches for cyclin D1-dependent cancers. Inhibition of PTC cell proliferation by CDK4/6 inhibition or broader targeting of cyclin D1 action by drugs that cause cyclin D1 downregulation or protein degradation.

If an important component of the oncogenic action of cyclin D1 is accomplished *via* CDK-independent mechanisms, anti-cyclin D1 may be expected to be especially efficacious in cyclin D1-driven PTCs. The effective use of such new approaches is centered on the tailored selection of PTC subgroups that are likely to respond to treatment. In PTC, high nuclear cyclin D1 levels are associated with aggressive clinicopathological features, including lymph node metastasis, tumor recurrence, extrathyroidal invasion, and more advanced initial tumor stages. As described, aberration of activated Wnt/β-catenin signaling pathway enhances the transcriptional activity of *CCND1* and increases nuclear accumulation of cyclin D1. Activation of MAPK and PI3K/AKT pathways, which is usually induced by BRAF and RAS family mutations, are the two most fundamental causative factors of PTC (121, 122). The RAS**–**MAPK pathway regulates cell cycle entry by upregulating cyclin D1 expression in PTC cells (56). Therefore, β-catenin, BRAF, and RAS may serve as useful biomarkers in patients requiring cyclin D1-based therapy for PTC. Patients receiving this therapy may have longer progression-free and a better overall survival than those receiving standard therapies that are accepted in cases of unsuccessful surgery and radioiodine therapy. However, targeting cyclin D1 is often considered difficult because of its lack of intrinsic enzymatic activity (11). Drug combination therapies that affect *CCND1* transcription or cyclin D1 degradation, target several end points of cyclin D1 function. Alternative methods may be employed to target cyclin D1 and block PTC progression (**Figure 2**).

Targeting of cyclin D1 using a combination of the retinoid X receptor (RXR) activator (Bexarotene) and a EGFR inhibitor (Erlotinib) has shown satisfactory levels of efficacy in clinical studies (123). Rosiglitazone diminishes the expression of cyclin D1, increases the expression of cell cycle inhibitors p21 and p27, and suppresses the proliferation and migration of tumor cells in a dose-dependent manner (124, 125). Both rosiglitazone and bexarotene exhibit anti-cancer activities against human PTC cells (126). The sodium/iodine symporter (NIS) is positively expressed in thyroid cancers with a good prognosis and plays a key role in response to radioiodine therapy. Moreover, NIS downregulation usually predicts tumor recurrence or treatment failure (126). In the setting of normal or hypoxic conditions, bexarotene synergizes with rosiglitazone to inhibit malignant cell growth and increase NIS levels in PTC, thereby demonstrating its potential as a chemotherapeutic candidate for PTC. However, despite their promising anti-proliferative effects, the efficacy and safety of rosiglitazone and bexarotene for PTC in humans require further verification.

Translation of CCND1 mRNA is dependent on mTOR, raising the possibility that mTOR inhibitors may hamper the cell cycle and tumor progression *via* cyclin D1 (101, 127). A recent clinical trial found, rapamycin, an mTOR inhibitor, to be therapeutically beneficial for nearly 40% of patients with advanced MCLs (128). An elaborate study demonstrated that the mTOR kinase inhibitor, CZ415, decreases cyclin D1 expression and induces G1-S arrest in human PTC cells. Moreover, it proved that CZ415 plays a role in the inhibition of cell proliferation and xenograft tumor growth in PTC (129). Thus, future studies on mTOR kinase inhibitor-based therapies may help improve the survival of patients with PTC.

Abnormal dysregulation of cyclin-CDK perturbs cell cycle control and allows continuous cell division (31, 130, 131). The earliest identified function of cyclin D1 was its role as a regulatory partner of CDK4/6 (11). Owing to increasing clinical applications of specific kinase inhibitors, a more direct and feasible method is to target cyclin D1 by inhibiting CDK4/6 (25, 132). Many first-generation CDK inhibitors have been excluded from research studies, partly because nonselective pan-CDK inhibition causes cytotoxicity in noncancerous cells (133, 134). However, these issues have been largely addressed by the newly developed CDK 4/6 pharmaceuticals with high specificity (132). Manageable cytotoxicity makes CDK 4/6 inhibitors (palbociclib, abemaciclib, and ribociclib) attractive candidates for anti-cancer drugs (135, 136). A key phase III trial of palbociclib for advanced ER-positive breast cancer showed improved progression-free as well as overall survival, with well tolerated toxicity. Testing for CDK4/6 inhibitors in low-risk patients with PTC might be difficult for ethical reasons. Extending the use of CDK4/6 inhibitors beyond ER-positive breast cancer is challenging and requires biomarkers that are predictive of the applicable standards. Antonello et al., demonstrated that targeting CDK4/6 in aggressive PTC helps overcome CDK4/6-dependent cell cycle checkpoint dysfunction and triggers apoptosis (137). Combination therapy with CDK4/6 inhibitors downregulates pAKT levels, resulting in changes in its downstream effectors. In addition, combination treatments with CDK4/6 inhibitors showed impressive improvements in the ability to overcome both primary and secondary resistance offered to treatments with single agents in invasive human PTC cell lines (137). Radioactive iodine (RAI) is administered for differentiated thyroid cancer (DTC) with intermediate to high-risk features (138). However, some patients with DTC and distant metastases exhibit dedifferentiation and decreased iodine uptake, rendering adjuvant treatment with RAI ineffective (138). Anaplastic thyroid carcinoma (ATC) is the most aggressive type of thyroid cancer. It accounts for a disproportionate number of thyroid cancer-related deaths, because of its resistance to current therapeutic approaches (138, 139). The selective CDK4/6 inhibitor, ribociclib, induces cell cycle arrest at the G0-G1 phase, facilitates cell apoptosis, and inhibits cell proliferation in ATC (140). Lack of expression or dysfunction of NIS is considered to be an important causative factor of iodine-resistant tumors (138). Studies have indicated that mutations in MAPK and/or PI3K (BRAF V600E and RAS) may decrease NIS expression by directly affecting its transcription (141, 142). BRAF V600E and MEK inhibitors can re-sensitize dedifferentiated thyroid cancer to iodine, thus allowing RAI treatment, leading to improved disease control (143, 144). The selective BRAF inhibitor (dabrafenib) can stimulate radioiodine uptake in patients with metastatic BRAF V600E mutant iodine-refractory PTC (143). Taken together, these studies suggest that BRAF V600E may serve as a promising biomarker for predicting high-risk radioiodine-resistant PTC, in patients enrolled for CDK4/6 inhibitor clinical trials. Specific CDK4/6 inhibitors have fewer therapeutic targets and are, therefore, more specific than multi-kinase inhibitors. Although, a clinical study of combined therapy with CDK4/6 inhibitors against PTC, is yet to be conducted, this approach may provide new insights to the possibility of improving the overall survival of patients with PTC and minimizing the risk of drug resistance and side effects.

## Conclusion

Since the discovery of *CCND1* in 1991, considerable progress has been made towards a better understanding of its physiology and role in various diseases. Cyclin D1 is frequently deregulated in PTC and serves as a biomarker of cancer phenotype and disease progression. In this review, we discuss recent studies that uncovered the detailed role of cyclin D1 in cell cycle control and specific changes in PTC cells. With due consideration given to the oncogenic role of cyclin D1 in PTC carcinogenesis, cyclin D1 shows promise as a potential therapeutic target against PTC. Through high-throughput screening techniques, it is possible to identify novel and effective cyclin D1 inhibitors from a diverse library of chemical compounds. Meanwhile, the effective use of potential therapies towards cyclin D1 or CDK4/6 will rely on the development of biomarkers for the therapeutic response. Considering that the efficacy of most targeted therapies is limited due to drug resistance, it is important to understand the mechanisms underlying resistance to cyclin D1 or CDK4/6 inhibition. Thus, further studies leading to the development of optimization methods that enhance the anti-cancer effects of cyclin D1 inhibitors in PTC are warranted.

## Author contributions

HD and WC conceived the study. WC wrote the manuscript. WC and D-JL participated in the figure production. WC, L-ZS, D-JL, LZ, M-MW, and HD improved the manuscript quality. All authors contributed to the article and approved the submitted version.
